# Nitrogen Use Efficiency Phenotype and Associated Genes: Roles of Germination, Flowering, Root/Shoot Length and Biomass

**DOI:** 10.3389/fpls.2020.587464

**Published:** 2021-01-20

**Authors:** Narendra Sharma, Vimlendu Bhushan Sinha, N. Arun Prem Kumar, Desiraju Subrahmanyam, C. N. Neeraja, Surekha Kuchi, Ashwani Jha, Rajender Parsad, Vetury Sitaramam, Nandula Raghuram

**Affiliations:** ^1^School of Biotechnology, Guru Gobind Singh Indraprastha University, Dwarka, India; ^2^ICAR Indian Institute of Rice Research, Hyderabad, India; ^3^ICAR Indian Agricultural Statistics Research Institute, New Delhi, India; ^4^Anant Cooperative Housing Society, Pune, India

**Keywords:** nitrogen use efficiency, N-response, phenology, phenotype, nutrients, rice, phenomics

## Abstract

Crop improvement for Nitrogen Use Efficiency (NUE) requires a well-defined phenotype and genotype, especially for different N-forms. As N-supply enhances growth, we comprehensively evaluated 25 commonly measured phenotypic parameters for N response using 4 N treatments in six indica rice genotypes. For this, 32 replicate potted plants were grown in the green-house on nutrient-depleted sand. They were fertilized to saturation with media containing either nitrate or urea as the sole N source at normal (15 mM N) or low level (1.5 mM N). The variation in N-response among genotypes differed by N form/dose and increased developmentally from vegetative to reproductive parameters. This indicates survival adaptation by reinforcing variation in every generation. Principal component analysis segregated vegetative parameters from reproduction and germination. Analysis of variance revealed that relative to low level, normal N facilitated germination, flowering and vegetative growth but limited yield and NUE. Network analysis for the most connected parameters, their correlation with yield and NUE, ranking by Feature selection and validation by Partial least square discriminant analysis enabled shortlisting of eight parameters for NUE phenotype. It constitutes germination and flowering, shoot/root length and biomass parameters, six of which were common to nitrate and urea. Field-validation confirmed the NUE differences between two genotypes chosen phenotypically. The correspondence between multiple approaches in shortlisting parameters for NUE makes it a novel and robust phenotyping methodology of relevance to other plants, nutrients or other complex traits. Thirty-Four N-responsive genes associated with the phenotype have also been identified for genotypic characterization of NUE.

## Introduction

Reactive nitrogen (N) is the largest nutrient input for agriculture, but the imbalanced, excessive or inefficient use of N fertilizers is increasingly becoming an economic as well as environmental burden, both globally (Sutton et al., 2019; Zhang et al., 2020) and in India (Abrol et al., 2017). Cereals consume most of the N fertilizer globally, especially the predominantly rice growing countries of Asia and Latin America (Zhang et al., 2015). Nitrogen use efficiency (NUE) is defined in many ways including uptake, utilization or photosynthetic efficiency, but agronomically understood as output–input ratio and expressed in cereals as grain yield per unit N input (Raghuram and Sharma, 2019). Rice is an ideal target crop to improve NUE because of its lowest NUE among cereals (Zhang et al., 2015), its huge genetic diversity that includes wild relatives and a vast post-genomic resource base. Urea is the predominant N fertilizer in most of the rice-growing developing countries, whereas nitrate is more commonly used in the developed world. Agricultural soils tend to have a mix of multiple forms of N (Akhtar et al., 2012), which have not yet been factored into by any biological studies to characterize NUE so far.

While some improvement in agronomic NUE can be attained through slow-release fertilizers (Li T. et al., 2018), bio-fertilizers (Bargaz et al., 2018; Quevedo-Amaya et al., 2020) and crop management practices (Guo et al., 2017; Cui et al., 2018; Santiago-Arenas et al., 2020), the improvement of inherent plant NUE has to be tackled biologically. The lack of a well-defined phenotype for N response and NUE has hampered biological interventions for crop improvement (Mandal et al., 2018; Raghuram and Sharma, 2019; Sinha et al., 2020). However, there has been some recent progress in identifying phenotypic parameters for N-response/NUE in model plants (Li et al., 2017). In rice, the parameters include leaf chlorophyll content (Ladha et al., 2007; Thind and Gupta, 2010; Mathukia et al., 2014; Jia et al., 2020), dense and erect panicle (Sun et al., 2014) and root length and density (Morita et al., 1988; Xu et al., 2012; Yang et al., 2012; Li et al., 2015; Peng et al., 2015; Rogers and Benfey, 2015; Kiba and Krapp, 2016; Steffens and Rasmussen, 2016; Xie et al., 2017; Duque and Villordon, 2019). But no study has comprehensively evaluated all the phenotypic parameters for N-response in urea and nitrate, nor distinguished between N-response and NUE phenotypically in any crop.

There have been several attempts to find genes involved in N-response through functional genomics (Pathak et al., 2020). Single gene transgenics were used to improve N-uptake, assimilation or remobilization, but with little improvement of NUE, if any (Mandal et al., 2018; Raghuram and Sharma, 2019; Sinha et al., 2020). Recent attempts in rice have been more encouraging (Fang et al., 2013; Fan et al., 2014; Sun et al., 2014), especially when NAR2.1 and NRT2.3a were co-overexpressed (Chen et al., 2020). However, there is ample scope to find newer regulatory targets outside the N-metabolic pathway, or revert to forward genetics approach of using phenotype. Even genome-wide association mapping (Tang et al., 2019) and marker development require proven phenotypic association data.

Recently, we showed that germination and crop duration constitute the NUE phenotype in rice and ranked 21 genotypes for yield at low N (Sharma et al., 2018). Earlier physiological studies in rice clearly linked seed weight, germination and yield: Larger/heavier seeds respired faster, germinated faster, flowered early and yielded less, indicating the overall meristematic control throughout the plant life cycle (Sitaramam et al., 2008). Since osmotica inhibit respiration universally, their effects at different stages of the Arabidopsis life cycle were used to confirm the energetic basis of phenological traits (Sitaramam and Atre, 2007). Vegetative plant growth, largely dominated by vacuolar elongation, is necessary but not sufficient for yield in cereals due to the involvement of reproductive parts in grain yield, unlike in green leafy vegetables (Löfke et al., 2015). Accordingly, the measurements of the effect of N on various phenotypic parameters may be logically classified into germination, vegetative and reproductive categories, to derive the most effective parameters that contribute to NUE.

We report here the most comprehensive study of NUE phenotype in any crop, based on life-long analysis of N-responsive changes in 25 parameters using six rice genotypes. We identified and shortlisted parameters that are specific to only N-response or only NUE or both, and field-validated a pair of contrasting genotypes for yield and NUE.

## Materials and Methods

### Plant Materials

Six rice genotypes of *Oryza sativa* ssp. indica were used, of which three were from the early germinating or short duration group *viz*, Aditya, Swarnadhan, and Nidhi and the other three were from the late germinating or long duration group, namely, Panvel1, Triguna, and Vikramarya. They were selected from our previous phenotypic ranking of 21 indica genotypes for NUE (Sharma et al., 2018). The seeds of the genotype Panvel1 were procured from Kharland rice research station, Panvel, Maharashtra, India, while the seeds of all other genotypes were from the ICAR Indian Institute of Rice Research, Hyderabad, India.

### Green-House Experimental Conditions

Seeds of the above six rice genotypes were weighed individually and only seeds of modal weight (+0.5 mg) were used for the green-house studies to control intra-varietal variation (Sitaramam et al., 2008; Sharma et al., 2018). They were surface-sterilized with 0.1% mercuric chloride for 50 s, followed by 8–10 washes with deionized water and soaked in deionized water for 2 h. They were then sown in pots filled to two-thirds of their height with nutrient-depleted sand, prepared by repeated washing till it had no detectable N as described earlier (Sharma et al., 2019). Prior to sowing, the pots were saturated for 2 days with Arnon–Hoagland medium (Hoagland and Arnon, 1950) containing nitrate or urea as the sole source of N at normal (15 mM N) or low level (1.5 mM N). It was achieved in the case of urea by replacing the dual sources of N [5 mM each of KNO_3_ and Ca (NO_3_)_2_] in the original medium with 7.5 mM urea to maintain the total N concentration at 15 mM. The media components were obtained from SRL, India. There were 32 replicate pots/plants for each of the six genotypes and 4 N treatments (low or 1.5 mM nitrate N, normal or 15 mM nitrate N, low or 0.75 mM urea N, and normal or 7.5 mM urea N). Therefore, the experimental design was 6 × 4 × 32 as shown in Supplementary Figure S1. The growth conditions in the green-house were 28°C temperature, 75% humidity, 270 μmol m-2 s^–1^ light intensity and 12/12 h photoperiod. The pots were replenished with sterilized media to saturation every 3 or more days as needed, till the excess media drained off from the perforated bottom of the pot.

The measured physiological and agronomic parameters for phenotyping were based on literature and IRRI-IBPGR (1980). They have been classified into germination (G), vegetative (V) and reproductive (R) categories, numbered for lucidity and their full forms and short forms italicized throughout for better readability. All the timed measurements were based on the number of days from sowing. They were: *weight of the individual seeds* and *days to germination* (G), which was measured as the day of visible emergence of plumule from the soil, 15 vegetative (V) and 9 reproductive (R) parameters *viz*, *chlorophyll content before flowering at 79 days* (V1: *chlorophyll*) measured by SPAD meter, *green leaf number per plant at vegetative stage at 81 days* (V2: *green leaves at vegetative stage*) counted manually, *yellow leaf number per plant at vegetative stage at 81 days* (V3: *yellow leaves at vegetative stage*) counted manually, *total leaf number per plant at vegetative stage at 81 days* (V4: *total leaves at vegetative stage*) counted manually, *leaf width before flowering at 86 days* (V5: *leaf width*) for which youngest leaf was used to measure in mm with the help of a scale, *stem thickness before flowering at 87 days* (V6: *stem thickness*) measured by Vernier calipers from the lower half of the stem, *shoot length before harvest* (V7) measured in mm from the base of the plant to the tip of the longest leaf of the plant every 10 days until it stabilized), *total leaf number per plant at the onset of flowering* (V8: *total leaves at flowering*) counted manually, *green leaf number per plant at the onset of flowering* (V9: *green leaves at flowering*) counted manually, *yellow leaf number per plant at the onset of flowering* (V10: *yellow leaves at flowering*) counted manually, *fresh biomass per plant without panicle at harvest* (V11: *fresh biomass*) in mg, *shoot length after harvest* (V12) measured in mm from root-shoot transition point to the tip of the longest leaf, *root length* (V13: *root length*) in mm from root-shoot transition point to the tip of the longest root, *total plant height (root* + *shoot) at harvest* (V14: *total plant height*) in mm from the longest root tip to the longest leaf tip, *dry biomass per plant (without panicle) at harvest* (V15: *dry biomass*) weighed in mg repeatedly after drying at 65°C in an oven, until it stabilized, *days to onset of flowering* (R1: *flowering*) counted manually from the date of sowing, *unfilled grain weight per plant* (R2) in mg *total grain weight per plant* (R3) in mg, *filled grain weight per plant* (R4) in mg, *panicle weight per plant* (R5) in mg, *filled grain number per plant* (R6) counted manually, *total grain number per plant* (R7) counted manually, *unfilled grain number per plant* (R8) counted manually, *weight of panicle remains per plant* (R9), measured in mg after removing seeds from panicles. The data for all vegetative parameters were collected between 79 and 87 days before flowering began in any of the six genotypes. For each parameter, data were collected in a single day across all 768 plants spanning all genotypes and N treatments. Out of the six genotypes, Swarnadhan did not set seed at all and therefore not included in the analysis. Thus, the final analysis of greenhouse data was based on 4 treatments × 5 genotypes = 20 combinations.

### Field Experimental Conditions

Two out of the six rice genotypes used for phenotyping in the greenhouse *viz*, Nidhi and Panvel1 were evaluated under field conditions at the ICAR Indian Institute of Rice Research for three seasons over 2 years (Kharif, 2017, 2018; Rabi, 2017-2018). The dates of sowing, planting and harvest are provided in Supplementary Table S1. The geographical coordinates of the experimental farm were 17° 19′′ N latitude and 78° 23′′ E longitude, at an altitude of 542 m above sea level and mean annual precipitation of 750 mm. The characteristics of the field soil were, pH 8.1; EC 0.7l dS/m; free CaCO_3_ 5.01%; CEC 44.1 C mol (p+)/kg soil; soil organic carbon 0.70%; soil available N 215 kg/ha; available phosphorus 46 kg/ha; potassium 442 kg/ha, and zinc 12.5 ppm.

The field experiment was conducted by transplanting 1 month old seedlings in a split plot design with plot size 9.0 m × 5.5 m (L × W) with N levels as the main plot and varieties as the subplots in two replicates for each of the three seasons. The cropping geometry was 20 cm × 10 cm (line to line distance × plant to plant distance), hence for every square meter, there were 50 hills. Urea was the sole source of applied N at the rate of 50 (N50), 100 (N100), 150 (N150) kg N/ha in three equal splits (1/3 at basal, 1/3 at tillering, and 1/3 at panicle initiation stage), with a control of no added N (N0). The same plots were used for N0, N50, N100, and N150 treatments in each season. Yield parameters *viz*, grain weight per meter square and thousand grain weight were measured using plants from a sample of one-meter square from two different locations of each plot, containing 50 plants per sample. For all other parameters 5 representative plants were taken per sample, so that each measurement yielded two replicate sample values, each representing five plants. This was repeated over three seasons so that there were six data points in all for every parameter (2 replicates per season × 3 seasons). They were harvested at maturity, divided into vegetative and reproductive parts, dried and weighed for determining dry matter. Grain and straw yield was adjusted to 14% grain moisture content and expressed in kg ha^–1^. Straw and grain samples were analyzed for N in a FOSS automatic nitrogen analyzer using the inbuilt micro Kjeldahl protocol. The various yield and NUE indices were calculated as per their standard definitions as follows: agronomic NUE (AgNUE, kg grain yield increase/kg N added; Yield in N treated plot – yield in control)/N applied), partial factor productivity (PFP, kg grain/kg N added; Grain Yield/N applied), internal efficiency (IE, kg grain/kg N taken up; Grain Yield/Total N uptake), physiological efficiency (PE, kg grain yield increase/kg N taken up; Yield in N treated plot – yield in control)/(N uptake in N treated plot- N uptake in control), recovery efficiency (RE, Increase in N uptake/unit N added; N uptake in N treated plot- N uptake in control)/N applied × 100) and nitrogen harvest index [NHI, N uptake grain/N uptake total (grain + straw)].

### Network and Feature Selection Analyses

To map the interactions between parameters, a correlation matrix was constructed using the mean values of each parameter for 32 replicates on each of the five genotypes under four N treatments (low/normal nitrate/urea). This was done separately for low or normal dose of N (data not shown as they were similar), as well as for the pooled data of both doses as shown. The significantly correlated parameters were used as source and target groups to cluster and construct interaction networks using Cytoscape ver. 3.6 (Shannon et al., 2003) with y files layout, in order to visualize the most connected N-responsive parameters that may together contribute to NUE. Separate networks were made for combined data of N, only nitrate and only urea.

To bioinformatically rank the phenotypic parameters based on their N-response, Feature selection was carried out using Java Machine Learning library (Abeel et al., 2009). As NUE is best understood in terms of yield, all the grain-related reproductive parameters that always ranked at the top were excluded to repeat the Feature selection analysis with the remaining 17 parameters (G, V, and R1) to identify parameters that contributed to NUE, other than those related to yield. The mean values of 17 parameters were used for 32 replicates of 5 rice genotypes under normal N (control) and low N (test) levels, for only nitrate, only urea, and combined N data.

### Data Analysis

MS excel was used to calculate coefficients of variation. For this purpose, the mean values of 32 replicates for each treatment were averaged for all five genotypes and used to determine the coefficients of variation using the formula [(standard deviation/mean)^∗^100] and plotted against the time of measurement. SPSS ver. 16 was used to perform ANOVA, MANOVA and Principal Component Analysis (PCA). ANOVA was done to identify parameters in which N effect was significant for each of the parameters separately. The % effect of normal N minus low N on each of the 25 phenotypic parameters was calculated using the mean values of 32 replicates and pooled for all the genotypes. MANOVA was done to identify whether N effect was significant when parameters were bunched into vegetative and reproductive groups. PCA was done to plot principal components 1 *vs*. 2 and 2 *vs*. 3 to identify the grouping of vegetative, reproductive and germination parameters. For this purpose, the mean values of 32 replicates for normal and low urea treatments were transformed using (X-mean)/STD and taken together for all five genotypes were used. These transformed mean values of each parameter were used for all genotypes and subjected to PCA separately for nitrate and urea treatments, by pooling their data for both doses (low/normal). Correlation coefficients between NUE and shortlisted parameters from Feature selection and network analysis were obtained and their significance was tested using MS Excel. Partial Least Squares Discriminant Analysis (PLS-DA) was carried out using the software tool SIMCA (trial ver. 15, Umetrics, Umea, Sweden) to revalidate the results obtained through Feature selection and significance of correlation coefficients.

### Meta Data Analysis

N responsive genes were mined from our microarray data of rice N-response in ssp. indica and ssp. japonica deposited at NCBI (GSE 12940, GSE20924, and GSE140257). Genes associated with the eight shortlisted phenotypic traits were retrieved from Oryzabase database^1^, while genes associated with yield were retrieved from database RicyerDB (Jiang et al., 2018) and literature. Venn selections were carried out to find common genes between different datasets using the online tool Venny2.1^2^. The protein sequences of genes were retrieved from RAP database and their predicted sub-cellular localizations was determined using the program Prot Comp 9.0 with default parameters for plants^3^. Rice Expression Database (Xia et al., 2017) was used to find the tissue/stage specific expression of selected genes.

## Results

### Reproductive Parameters Have Higher Variance Between Genotypes and N Forms/Doses

**FIGURE 1:**
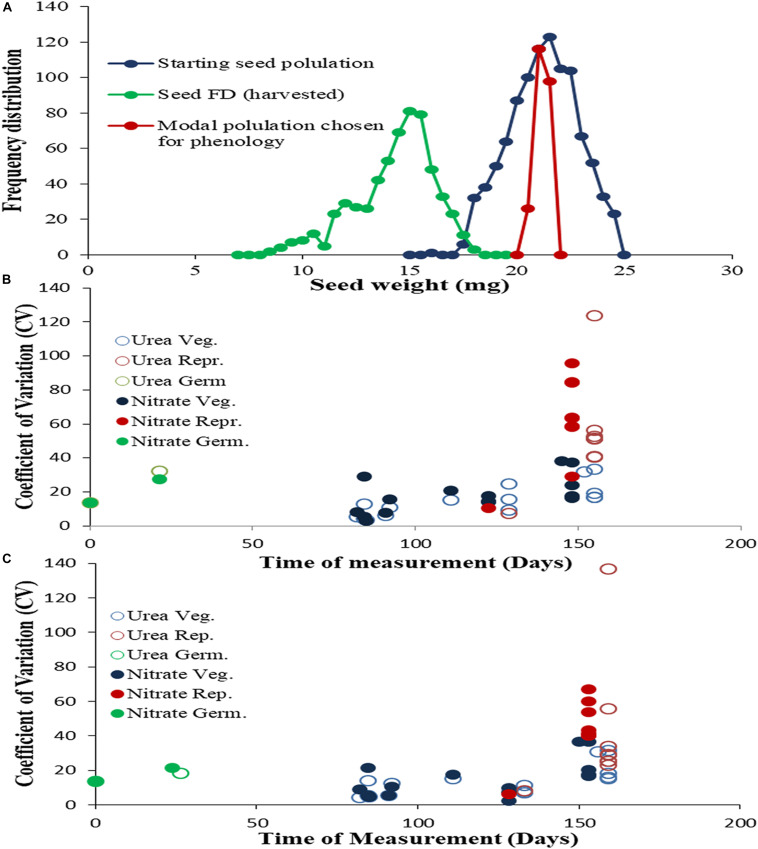
Magnitude of variation in phenotypic parameters throughout the life cycle: **(A)** Frequency distribution of individual seed weights for the starting seed population of the genotype Nidhi and their subset of modal weight used for sowing, as well as for harvested seeds: First generation seeds (Blue), *n* = 1000, weight 21.01 ± 1.6 mg, skewness, –0.131, kurtosis –0.54; modal seeds Green, *n* = 240, weight 20.9 ± 0.28 mg, skewness –0.058, kurtosis, –1.203, Second generation seeds (Red), *n* = 600, weight, 13.9 ± 2.3 mg, skewness, –1.717, kurtosis 4.813). N response in the first generation was measured for each of the 25 phenotypic parameters using 32 replicate potted plants of five rice genotypes at **(B)** normal N (15 mM) and **(C)** low N (1.5 mM) of either urea or nitrate as the sole source of N. The mean values of 32 replicates for each treatment were averaged for all five genotypes and used to determine the coefficients of variation using the formula [(standard deviation/mean)*100] and plotted against the time of measurement. The parameters have been grouped and color-coded as related to germination (green) vegetative (blue) and reproductive (red) growth, with open circles indicating urea treatments and filled circles indicating nitrate treatments.

Understanding the nature and extent of variation provides a robust basis for phenotyping. To assess the physiological variance between genotypes while controlling variance within the population of each genotype, the weight distribution of the starting seeds was recorded and only seeds of modal weight (+0.5 mg) were planted. Yet, the variance and normal weight distribution fully reappeared in the second-generation seeds (Figure 1A), indicating a genetically enforced restoration of variation. Further details on the nature and magnitude of phenotypic variation in N-response among genotypes throughout their life cycle were revealed by plotting the coefficients of variation of each parameter, against the time of measurement for normal N (Figure 1B) or low N (Figure 1C). The coefficients ranged from 2 to 38 for vegetative parameters and 6 to 136 for reproductive parameters, clearly indicating that physiological variation increased developmentally, from vegetative to reproductive stage. In terms of N-source and dose, the vegetative parameters showed higher variation in nitrate than in urea, with their coefficients ranging between 2–38 and 3–33 respectively, within which the variation also increased from low to normal N dose. However, the reproductive parameters showed higher variation in urea than in nitrate, with their coefficients ranging between 7–136 and 6–95 respectively. These results clearly demonstrate the differential effects of N form and dose on different aspects of growth physiology in the germplasm evaluated.

### Principal Component Analysis Segregates Vegetative and Reproductive Parameters

**FIGURE 2:**
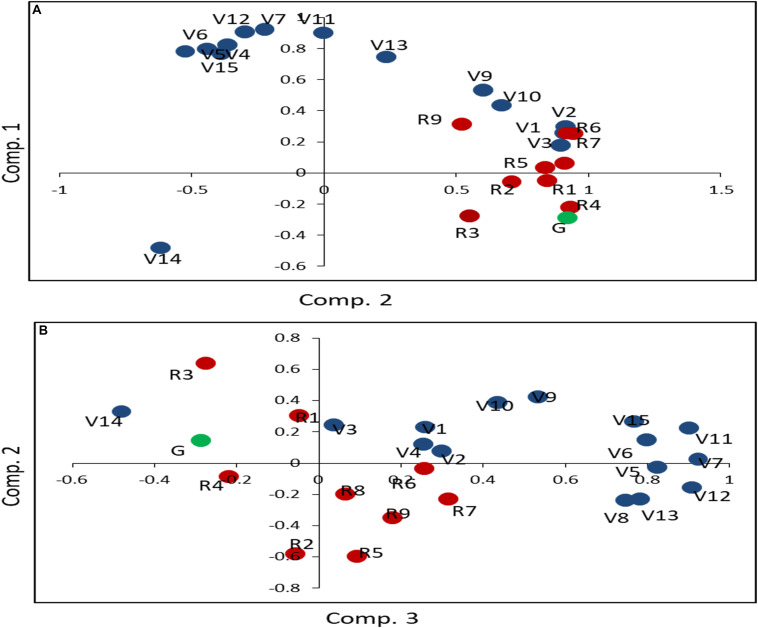
Principal component analysis of the effect of N on 25 phenotypic parameters in five rice genotypes. The mean values of 32 replicates for normal and low urea treatments were transformed using (X-mean)/STD and taken together for all five genotypes for principal component analysis. As the first three components accounted for 85% of the variance, plots of the principal component 1 vs. 2 **(A)** and 2 vs. 3 **(B)** have been shown. The parameters have been grouped and color-coded as related to germination (green) vegetative (blue) and reproductive (red) growth to denote: chlorophyll (V1), green leaf number at vegetative stage (V2), yellow leaf number at vegetative stage (V3), total leaf number at vegetative stage (V4), leaf width (V5), stem thickness (V6), shoot length before harvest (V7), total leaf number at flowering (V8), green leaf number at flowering (V9), yellow leaf number at flowering (V10), fresh biomass (V11), shoot length after harvest (V12), root length (V13), total plant height (V14), dry weight of plant (V15), flowering (R1), unfilled grain weight (R2), total grain weight (R3), filled grain weight (R4), panicle weight (R5), filled grain number (R6), total grain number (R7), unfilled grain number (R8), weight of panicle remains (R9). The segregation of these groups as apparent in these plots for urea treatment has also been found in nitrate treatment (data not shown).

To validate the qualitative differences in N-response and identify their groups statistically, PCA was carried out using transformed mean data of each parameter for all treatments for each genotype (Figure 2). The cumulative amount of phenotypic variation explained by the first three PCs was 84.66% (Supplementary Table S2). PC1 explained 47.87% of the phenotypic variation, with all the reproductive parameters and the vegetative parameters of *plant biomass, flowering*, and *germination* emerging as important within the characteristic vector of PC1. PC2 explained 27.62% of the phenotypic variation. Except V3, R1, R2, R5, R7, and R8, all other parameters emerged as important within the characteristic vector of PC2, comprising of *root/shoot length and plant biomass*. Overall, the plots for the first two principal components (Figure 2A) and between the 2nd and 3rd principal components (Figure 2B) clearly revealed that barring very few exceptions, there was a broad segregation of the vegetative (V) and reproductive (R) parameters, with germination (G) grouping with the latter.

### Magnitude and Significance of N-effect Varies on Different Phenotypic Parameters

**FIGURE 3:**
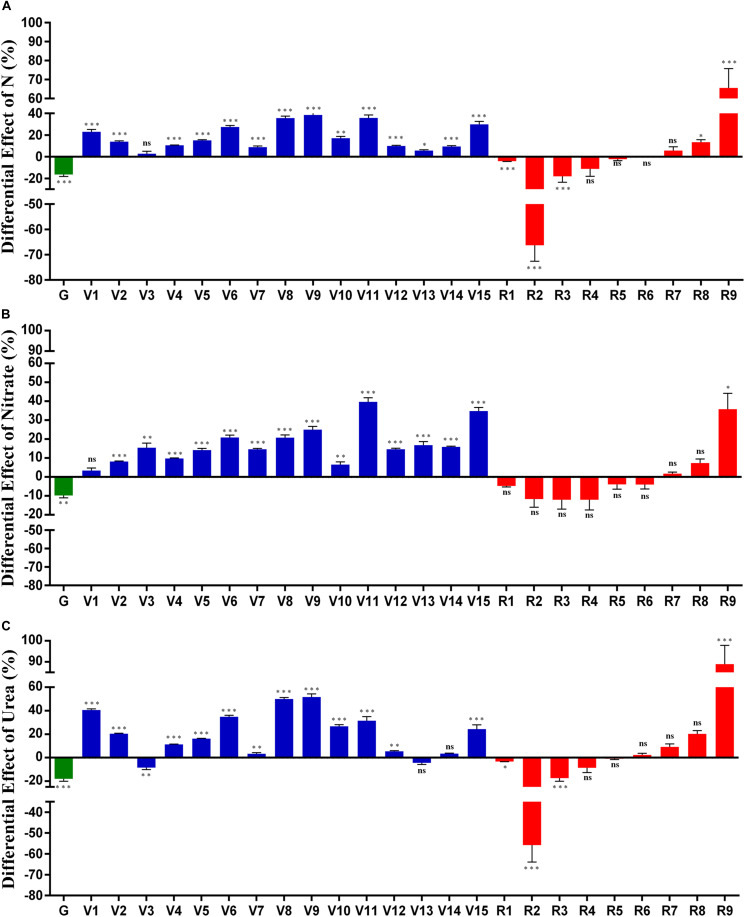
Differential effect of N on various phenotypic parameters. Seeds of modal weight from five genotypes were sown on nutrient-free sand in 32 pots for each genotype/N treatment and grown at 28°C in the green-house. They were fertilized with Arnon Hoagland medium with normal N (15 mM) or low N (1.5 mM) using urea or nitrate as the sole source of N. The % effect of normal N minus low N on each of the 25 phenotypic parameters was calculated using the mean values of 32 replicates and pooled for all the genotypes. The % effect of N is shown for **(A)** Combined data for nitrate and urea; **(B)** nitrate only and **(C)** urea only. The actual values of % N effect for each of the 25 parameters are shown in the table below the X axis of each plot. The measured parameters were grouped and numbered as germination-related (green) vegetative (blue) and reproductive (red) to denote: chlorophyll (V1), green leaf number at vegetative stage (V2), yellow leaf number at vegetative stage (V3), total leaf number at vegetative stage (V4), leaf width (V5), stem thickness (V6), shoot length before harvest (V7), total leaf number at flowering (V8), green leaf number at flowering (V9), yellow leaf number at flowering (V10), fresh biomass (V11), shoot length after harvest (V12), root length (V13), total plant height (V14), dry weight of plant (V15), flowering (R1), unfilled grain weight (R2), total grain weight (R3), filled grain weight (R4), panicle weight (R5), filled grain number (R6), total grain number (R7), unfilled grain number (R8), weight of panicle remains (R9). All the parameters have been arranged according to their time of measurement. Their significance values are indicated as asterisks for ^∗^*P* < 0.05, ^∗∗^*P* < 0.01, and ^∗∗∗^*P* < 0.001, respectively. All the values were significant (*P* < 0.05, ANOVA) except for R4, R5, R6, and R7 in plot A, V1, R1, R2, R3, R4, R5, R6, R7, R8 in plot B, V13, V14, R4, R5, R6, R7, and R8 in plot C.

Multivariate analysis of variance (MANOVA) with vegetative and reproductive parameters revealed that the effect of N was highly significant (*P* < 0.0001), regardless of whether the contrast was for the N form (nitrate or urea) or dose (normal, 15 mM and low, 1.5 mM). However, two-way ANOVA for N-treatments and genotypes revealed more nuanced effects of N on the individual parameters as elaborated chronologically in Figure 3 in terms of the % effect of normal N over low N for (A): Combined N data for nitrate and urea; (B): nitrate only and (C): urea only. Their mean data with their standard deviation and standard error are provided in Supplementary Tables S3, S4. For this purpose, mean data values for each of the parameters were averaged for all the genotypes and the % effect of N on each parameter (normal N minus low N) was plotted. It is evident that N affects different parameters to different extents, both in terms of N form and N dose. When the % effects of urea or nitrate are taken together (Figure 3A), the % effect of increasing N dose was positive on most of the vegetative (V) parameters by 2.7–38.4%. However, it was negative on most of the reproductive (R) parameters, by −0.22% to −66% and on *days to germination* (G) by −16.3%. They were all significant (*P* < 0.05, ANOVA), except R4, R5, R6, and R7. Overall, it is evident that N dose hastened germination and flowering, up-regulated most vegetative parameters and down-regulated most reproductive parameters (Figure 3A). The significantly up-regulated ‘reproductive’ parameters were *unfilled seed number* (R8, 13.39%) and the *weight of panicle remains* (R9, 65.5%), which could well be the vegetative part of a reproductive organ. The most down-regulated parameter was *unfilled grain weight* (R2, −66.2%).

The effect of nitrate alone and urea alone (Figures 3B,C) broadly resemble the overall effect of N, in terms of the opposite effects of boosting vegetative and limiting reproductive parameters. This was particularly true for nitrate, which also showed a broadly increasing effect of N from early to late vegetative development (Figure 3B). However, majority of the R parameters other than R9 were not significant in nitrate, whereas in urea, more R parameters were significant namely, *flowering* (R1), *unfilled grain weight* (R2), *total grain weight* (R3), and *weight of panicle remains* (R9, Figure 3C). The effect of nitrate dose on vegetative (V) parameters ranged between 3.4 and 39.7%, while the urea effect ranged between 3.3 and 51.6%, except for V3 and V13, on which urea had a negative effect. The most up-regulated parameter was *weight of panicle remains* (R9, 88.97% in urea) and *fresh biomass* (V11, 39.71% in nitrate), while the most down-regulated parameter was *unfilled seed weight* in urea (R2, −56.78%) and *filled grain weight* in nitrate (R4, −12.07%). These effects were significant (*P* < 0.05, ANOVA) for all the parameters except V1 and R1–R8 in nitrate (Figure 3B).

The effect of urea was broadly similar to that of nitrate, except on *yellow leaves at vegetative stage* (V3) and *root length* (V13) (Figure 3C). All the four most down-regulated parameters and two of the four most up-regulated parameters were the same between nitrate and urea treatments, though the extents of their regulation varied (Figures 3B,C). However, there were some differences in the manner in which nitrate and urea affected *panicle weight* (R5), *filled grain number* (R6), *total grain number* (R7), and *root length* (V13) (Figures 3B,C), which explain why different parameters appear to be non-significant in different N regimes.

### Network Analysis Reveals Highly Connected Phenotypic Parameters for N-response/NUE

**FIGURE 4:**
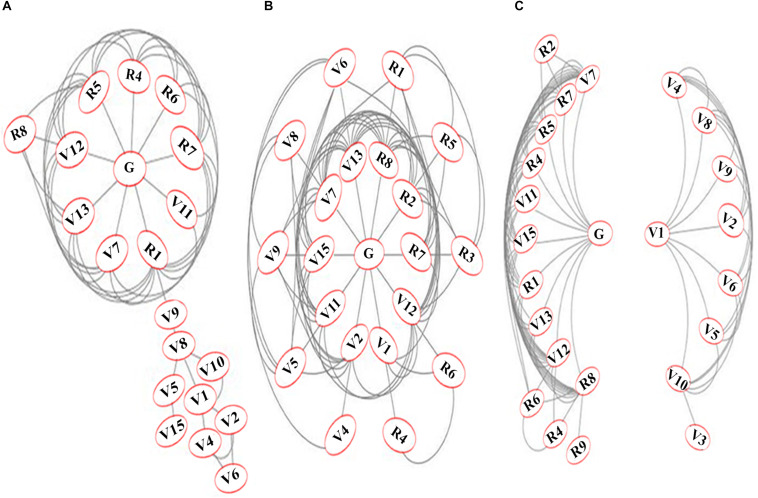
Networks of significantly correlated N-responsive phenotypic parameters. The networks were constructed using Cytoscape on the mean values of each parameter for 32 replicates on five genotypes and 4 N treatments (normal and low nitrate or urea). The networks are shown for **(A)** combined data for nitrate and urea; **(B)** nitrate only and **(C)** Urea only. The 25 measured parameters were grouped and numbered as germination-related (G) vegetative (V) and reproductive (R) to denote: chlorophyll (V1), green leaf number at vegetative stage (V2), yellow leaf number at vegetative stage (V3), total leaf number at vegetative stage (V4), leaf width (V5), stem thickness (V6), shoot length before harvest (V7), total leaf number at flowering (V8), green leaf number at flowering (V9), yellow leaf number at flowering (V10), fresh biomass (V11), shoot length after harvest (V12), root length (V13), total plant height (V14), dry biomass (V15), flowering (R1), unfilled grain weight (R2), total grain weight (R3), filled grain weight (R4), panicle weight (R5), filled grain number (R6), total grain number (R7), unfilled grain number (R8), weight of panicle remains (R9).

When multiple phenotypic parameters contribute to NUE, those that interact most may be better correlated among themselves than the rest. Such interactions were visualized as networks by correlation and clustering approach using Cytoscape software. Figure 4 shows the networks for 20 significantly correlated parameters for combined data of nitrate and urea (A), 21 significantly correlated parameters for nitrate only (B) and 24 significantly correlated parameters for urea only (C). In the combined network for nitrate and urea (Figure 4A), *flowering* (R1) emerged as the most connected parameter interacting with 10 of the 19 parameters. It was followed by *germination* (G), *panicle weight* (R5), and *total grain number* (R7) with nine connections, *shoot length after harvest* (V12, eight connections), *root length* (V13, seven connections), *filled grain weight* (R4, six connections), *shoot length before harvest* (V7, six connections), *filled grain number* (R6, five connections) and so on.

The nitrate network (Figure 4B) revealed *germination*(G) and *shoot/root length* (V12, V13) as the most connected, interacting with 12 of the 21 significant parameters for nitrate response. They were followed by 11 connections for *unfilled grain number* (R8) and *shoot length before harvest* (V7), 10 for *dry biomass* (V15), 9 for *fresh biomass* (V11) and so on, with 7 for *flowering* (R1). The urea network revealed two clusters, broadly separating the vegetative (V) and reproductive (R) parameters (Figure 4C), mainly because of lack of interaction between G and V1 as well as between R parameters and the majority of V parameters. The four vegetative parameters that clustered along with the reproductive parameters later turned out to be important for NUE, due to their strong correlation with yield. Interestingly, the most connected parameters of all three networks are same as those in the left cluster of the urea network. They reveal the hitherto unknown universal biological interactions/mechanisms underlying N-response and/or NUE.

### Shortlisting Phenotypic Parameters for N-responsive Yield and NUE

As NUE in rice is best measured as reproductive yield per unit N input and the relevant R parameters are inherent to the calculation of NUE, other parameters contributing to it from germination upto the flowering stage are of main interest for phenotyping. Feature selection analysis was used to rank 17 such parameters (Supplementary Table S5) using combined nitrate and urea data (A), only nitrate (B), or only urea (C). It is evident that *germination* (G), *flowering* (R1), *shoot length/plant height* (V12, V14) and *chlorophyll* (V1) ranked among the top half of parameters in all three N regimes. Three more parameters emerged when any two out of three N treatments were considered *viz*, *shoot/root length* (V7, V13) and *total leaves at flowering* (V8). Three more parameters ranked among the top 11 when any one of the three N treatments were considered *viz*, *fresh biomass* (V11), *dry biomass* (V15), and *green leaves at flowering* (V9). This allowed the shortlisting of 11 parameters: *germination* (G), *flowering* (R1), *chlorophyll* (V1), *shoot length before harvest* (V7), *total leaves at flowering* (V8), *green leaves at flowering* (V9), *fresh biomass* (V11), *shoot length after harvest* (V12), *root length* (V13), *total plant height* (V14) and *dry biomass* (V15). This shortlisting eliminated 6 parameters: *green leaves at vegetative stage* (V2), *yellow leaves at vegetative stage* (V3), *total leaves at vegetative stage* (V4), *leaf width* (V5), *stem thickness*, (V6), *and yellow leaves at flowering* (V10). Thus, 11 out of 17 parameters were shortlisted by this analysis for further validation.

### Correlation Analysis Validates Shortlisted Phenotypic Parameters for Grain Yield in Low N

To further validate and shortlist the 11 phenotypic V parameters for their contribution to yield as a derivative for NUE, their correlation with mean grain yield was examined in normal and low N. Each row of Supplementary Figure S2 represents one shortlisted parameter and each column represents either combined data for nitrate/urea, only nitrate or only urea. In the case of combined N data, nine parameters were significantly correlated for grain yield, especially at low N *viz*, *germination* (G), *flowering* (R1), *shoot length* (V7, V12) *total leaves at flowering* (V8), *biomass* (V11, V15), *root length* (V13), and *plant height* (V14). Thus, correlation analysis for combined N data validated nine out of 11 parameters shortlisted by Feature selection.

In the case of nitrate, six of the above nine parameters [except *flowering* (R1), *total leaves at flowering* (V8), and *root length* (V13)] correlated significantly for grain yield at low N level but were not significant under normal N levels (Supplementary Figure S2, middle column). In urea, eight of the above nine parameters (except *total leaves at flowering*, V8) correlated significantly with yield under low urea (Supplementary Figure S2, right column). Thus, separate correlation analyses on nitrate and urea treatments validated eight of the 11 parameters shortlisted by Feature selection.

### Shortlisted Phenotypic Parameters Correlated With NUE

To check whether the nine phenotypic parameters shortlisted above (by grain yield at low N) are a true reflection of NUE, their coefficients of correlation were calculated directly with NUE (grain yield/N). Eight of them significantly correlated with NUE in low nitrate/urea combined data *viz*., *germination* (G), *shoot length before harvest* (V7), *fresh biomass* (V11), *shoot length after harvest* (V12), *root length* (V13), *total plant height* (V14), *dry biomass* (V15), and *flowering* (R1) (Table 1 and Supplementary Figure S3, 1st column). Six of them also correlated significantly in low nitrate alone (Table 1 and Supplementary Figure S3, middle column), while all eight of them correlated significantly in low urea alone (Table 1 and Supplementary Figure S3, right column).

**TABLE 1:** Correlation of 9 shortlisted phenotypic parameters with NUE: NUE was calculated as grain yield/per unit N input for nitrate and/or urea.

**Parameters shortlisted for NUE phenotype**	**Normal nitrate and urea (combined data)**	**Low nitrate and urea (combined data)**	**Normal nitrate**	**Low nitrate**	**Normal urea**	**Low urea**
Germination (G)	NS	0.05	NS	0.05	NS	0.05
Chlorophyll (V1)	0.05	NS	NS	NS	0.01	NS
Shoot length before harvest (V7)	NS	0.001	NS	0.001	NS	0.001
Fresh biomass (V11)	NS	0.05	NS	0.01	NS	0.05
Shoot length after harvest (V12)	0.05	0.001	NS	0.001	0.01	0.001
Root length (V13)	NS	0.01	NS	NS	NS	0.05
Total plant height(V14)	0.05	0.001	NS	0.001	0.02	0.001
Dry biomass (V15)	NS	0.05	NS	0.01	NS	0.05
Flowering (R1)	0.05	0.01	NS	NS	NS	0.01

*(Total N input was calculated from all the doses of media added to the pots throughout the plant life cycle). Correlation coefficients between NUE and nine of the shortlisted parameters were determined in normal N and low N and the significance values are shown for either combined data for nitrate and urea, nitrate only and urea only.*

### Segregation of Phenotypic Parameters for NUE From N-response and Grain Yield

Out of the 25 phenotypic parameters tested for N-response to nitrate/urea, ANOVA revealed that 20 were significantly N-responsive, 13 of which were common for nitrate/urea, 16 in nitrate alone and 18 in urea alone (Table 2). Out of them, 11 were highly ranked by Feature selection and were also highly connected in network analysis. To segregate the NUE parameters from N-responsive parameters, nine of them that were significantly correlated with grain yield at low N were identified (Supplementary Figure S2). Their direct correlation with NUE revealed 8 parameters, viz., *germination* (G) *flowering* (R1), *shoot length* (V7, V12), *fresh* and *dry biomass* (V11, V15), *root length* (V13), and *plant height* (V14) (Table 2 and Supplementary Figure S3). Among them, V13 and R1 were significant for NUE only in urea, whereas the remaining six were significant in both nitrate and urea. Thus, these six emerged as global phenotypic parameters for N-responsive yield and NUE regardless of N regime (nitrate or urea). This is the segregation of few NUE parameters from 20 N-responsive parameters as well as their segregation in terms of nitrate/urea.

**TABLE 2:** Segregation and shortlisting of phenotypic parameters for N-response, yield and NUE: The % effect of normal N over low N on each of the 25 parameters was calculated using the mean values of 32 replicates and combined for all the genotypes.

**S. No.**	**N-responsive parameters**	**Low N-responsive yield related parameters**	**N- use efficiency parameters**
1	^(a,b,c)^ Germination (G)	^(a,b,c)^ Germination (G)	^(a,b,c)^Germination (G)
2	^(a,c)^ Chlorophyll (V1)	^(a,b,c)^ Shoot length before harvest (V7)	^(a,b,c)^Shoot length before harvest (V7)
3	^(a,b,c)^ Green leaf number at vegetative stage(V2)	^(a)^ Total leaf number at flowering (V8)	^(a,b,c)^Fresh biomass (V11)
4	^(b,c)^ Yellow leaf number at vegetative stage (V3)	^(a,b,c)^Fresh biomass (V11)	^(a,b,c)^Shoot length after harvest (V12)
5	^(a,b,c)^Total leaf number at vegetative stage (V4)	^(a,b,c)^Shoot length after harvest (V12)	^(a,c)^Root length(V13)
6	^(a,b,c)^Leaf width(V5)	^(a,b,c)^Root length(V13)	^(a,b,c)^Total plant height (V14)
7	^(a,b,c)^Stem thickness (V6)	^(a,b,c)^Total plant height (V14)	^(a,b,c)^ Dry biomass (V15)
8	^(a,b,c)^Shoot length before harvest (V7)	^(a,b,c)^Dry biomass (V15)	^(a,c)^Flowering (R1)
9	^(a,b,c)^Total leaf number at flowering (V8)	^(a,c)^Flowering (R1)	
10	^(a,b,c)^Green leaf number at flowering (V9)		
11	^(a,b,c)^Yellow leaf number at flowering (V10)		
12	^(a,b,c)^Fresh biomass (V11)		
13	^(a,b,c)^Shoot length after harvest (V12)		
14	^(a,b)^Root length (V13)		
15	^(a,b)^Total plant height(V14)		
16	^(a,b,c)^ Dry biomass (V15)		
17	^(a,c)^Days to flowering (R1)		
18	^(a,c)^Unfilled grain weight (R2)		
19	^(a,c)^Total grain weight (R3)		
20	^(ns)^Filled grain weight (R4)		
21	^(ns)^Panicle weight (R5)		
22	^(ns)^Filled grain number (R6)		
23	^(ns)^Total grain number (R7)		
24	^(a)^Unfilled grain number (R8)		
25	^(a,b,c)^Weight of panicle remains (R9)		

*Twenty of the 25 parameters were confirmed as N-responsive based on the significance test and shown in the 1st column. Ten of these parameters shortlisted from Feature selection and network analyses were subjected to correlation analysis with grain yield in normal and low N and those parameters that significantly correlated with yield at low N are shown in the 2nd column. NUE was calculated as yield per unit N input for all genotypes/treatments and their correlation with each of these 10 parameters was analyzed. Those that significantly correlated with NUE are shown in the 3rd column. The superscripted alphabet a/b/c over the parameter I first column denotes parameter in combined nitrate and urea N, only nitrate N and only urea N.*

### PLS-DA Validates the Shortlisted NUE Parameters by Clustering Them With Yield Parameters

In order to further validate the results obtained through PCA, Feature selection and significance of correlation coefficients, an independent partial least square discriminant analysis (PLS-DA) was done. For this purpose, we used the mean values of N-response for all 25 parameters in low or normal doses of nitrate or urea or both. It clustered all the above shortlisted NUE parameters along with all the reproductive yield parameters and clearly separated all the others (Supplementary Figure S4), independently validating those shortlisted for NUE. Interestingly, the few vegetative parameters that clustered along with the reproductive parameters later turned out to be important for NUE, due to their strong correlation with yield.

### Novel and Robust Methodology Relies on Correspondence Between Multiple Approaches

**FIGURE 5:**
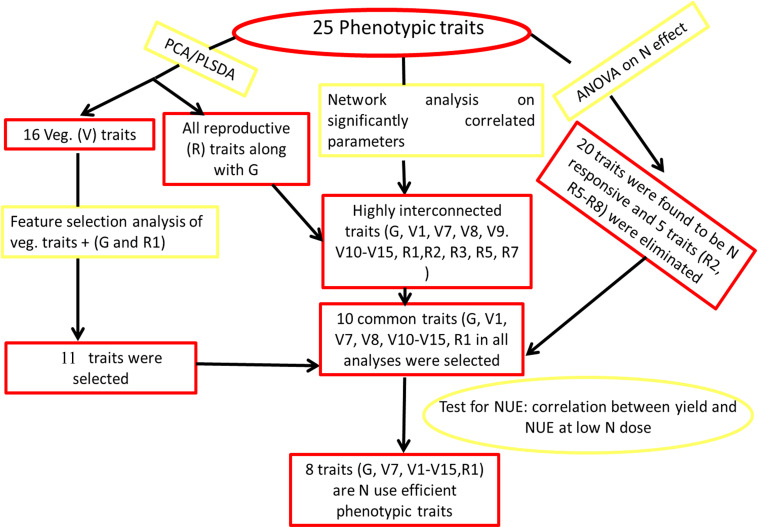
Potential methodology for shortlisting of phenotypic parameters. Various tools shown here can be used in the mentioned sequence to find out the phenotypic parameters for nitrogen use efficient or any other trait.

Our approach to data analysis in this study combined several commonly used statistical tools such as PCA, PLS-DA, and ANOVA with novel application of computational tools in NUE phenotyping, such as Feature selection and networks. Figure 5 summarizes them as a novel methodological flowchart: Both PCA (Figure 2) and PLS-DA (Supplementary Figure S4) independently segregated all the 25 measured parameters into very similar vegetative and reproductive groups, with germination clustering with the latter. Similarly, ranking of vegetative parameters, *germination* and *flowering* by Feature selection converged with 10 phenotypic parameters shortlisted based on the network analysis of significantly correlated parameters. These 10 were a subset of the 20 significantly N-responsive parameters identified by ANOVA (Figure 3 and Table 2). Most significantly, eight out of these 10 parameters correlated very significantly with NUE in terms of yield per unit N (Tables 1, 2 and Supplementary Figure S3). This convergence between multiple approaches of data analysis makes it a robust phenotyping methodology for NUE and possibly other quantitative traits.

### Field Data Validate NUE in Two Genotypes

**FIGURE 6:**
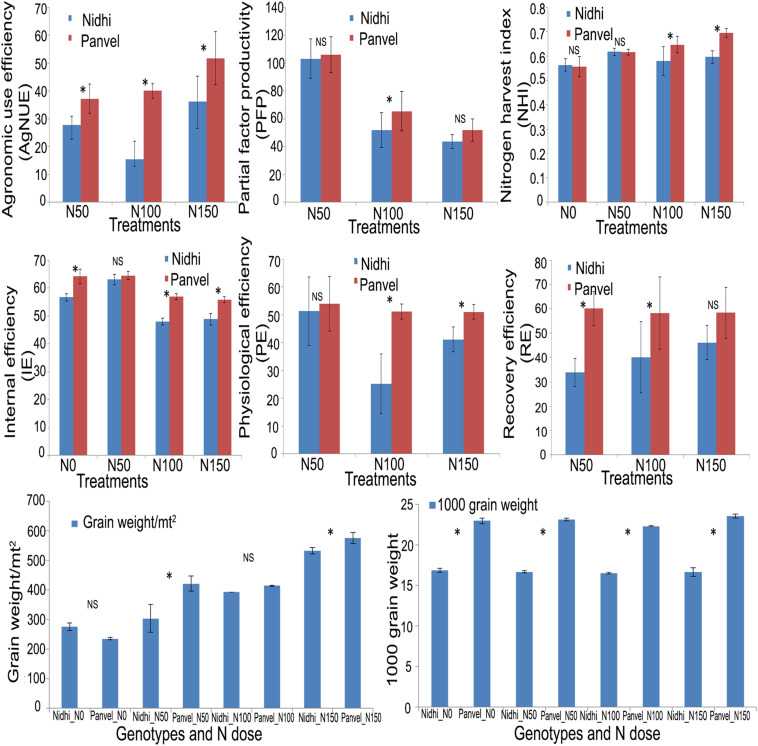
Field validation of the performance of rice genotypes. Field evaluation of the genotypes Nidhi and Panvel1 was conducted for 3 seasons over 2 years in N0, N50, N100, and N 150 N kg of added urea/ha. Mean data of the effect of urea dose on the genotypes are shown for grain weight/m^2^, 1000 grain weight, recovery efficiency, partial factor productivity, nitrogen harvest index and harvest index. The significance levels by *t*-test are shown between the bars being compared (* denotes significant at *P* < 0.05, and ns denotes non-significant).

The field performance of two of the six rice genotypes originally used for phenotyping in this study (Nidhi and Panvel1) was evaluated over three seasons using four treatments – either no added N (N0) or three different doses of urea (50, 100, and 150 kg N/ha). The late-germinating, late-flowering, long duration genotype Panvel1 performed significantly better (*P* < 0.05) than the early germinating, early flowering, short duration genotype Nidhi, in terms of the yield and NUE parameters measured *viz*., grain yield (g/m^2^), 1000 grain weight, recovery efficiency, partial factor productivity, physiological efficiency, internal efficiency and agronomic efficiency except nitrogen harvest index, which was only significant at N100 and N150 but not at N0 and N50 (Figure 6).

### Metadata Analyses Point to the Molecular Basis for NUE Phenotype

In order to understand the molecular basis of the phenotype for NUE, literature and online databases were explored as described under “Metadata Analysis” in section “Materials and Methods.” A search at Oryzabase yielded 105 genes associated with any of the eight phenotypic traits shortlisted for NUE in this study, as shown in the last column of Table 2. These 105 genes listed in Supplementary Table S6 include some genes from mitochondria and chloroplast. Their predicted protein localization revealed a different distribution, such as 35% in the extracellular region, followed by 26% in the nucleus, 14% in the plasma membrane, 10% in the chloroplast and 3% in the mitochondria (Supplementary Figure S5). These results indicate that apart from the nucleus, organelles might play a significant role in the regulation of the NUE phenotype.

**FIGURE 7:**
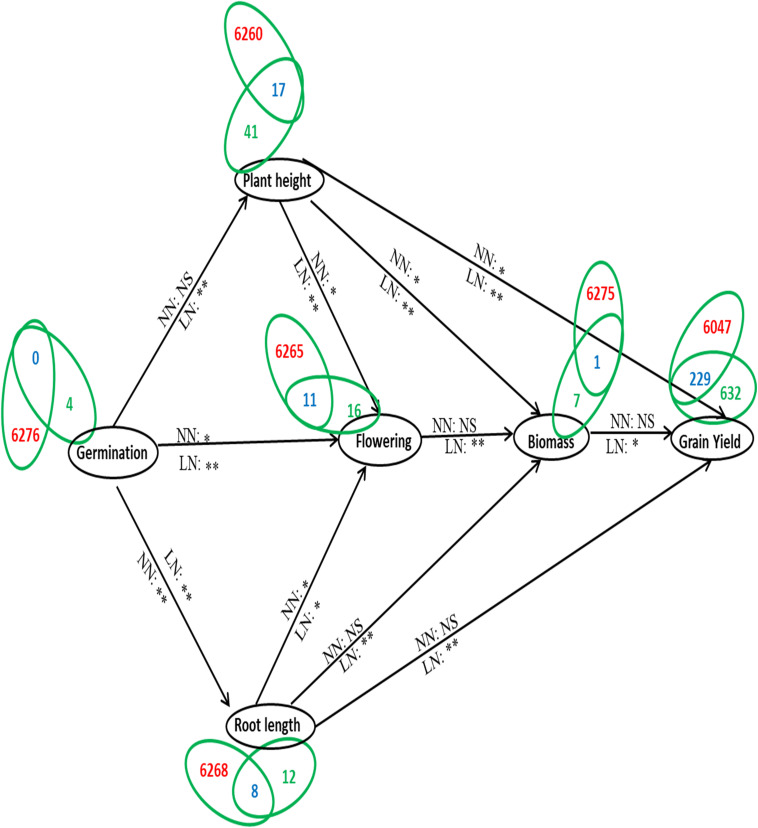
A model depicting the NUE phenotype in terms of molecular physiology. The shortlisted parameters constituting the NUE phenotype are depicted in terms of the growth physiology of the plant, they are germination, plant height, root length, flowering, biomass, and grain yield. The alphabets NN (normal urea or 15mM N) and LN (low urea or 1.5 mM N) along the arrows denote the N treatments for which correlation were calculated between connected phenotypic parameters along with the significance values of their urea response (* denotes significant at *P* < 0.05, ** denotes significant at *P* < 0.01, and ns denotes non-significant). The number of genes involved in each phenotypic parameter is indicated by Venn selections between the 6276 N-responsive genes compiled from NCBI GEO/database (GSE 12940, 20924, 140257) and the number of genes reported to be associated with each phenotypic parameter. The numbers in red denote the genes specific to N-response while those in green denote genes related to that particular phenotypic parameter and blue denotes the genes common between nitrogen and the phenotypic parameter.

**FIGURE 8:**
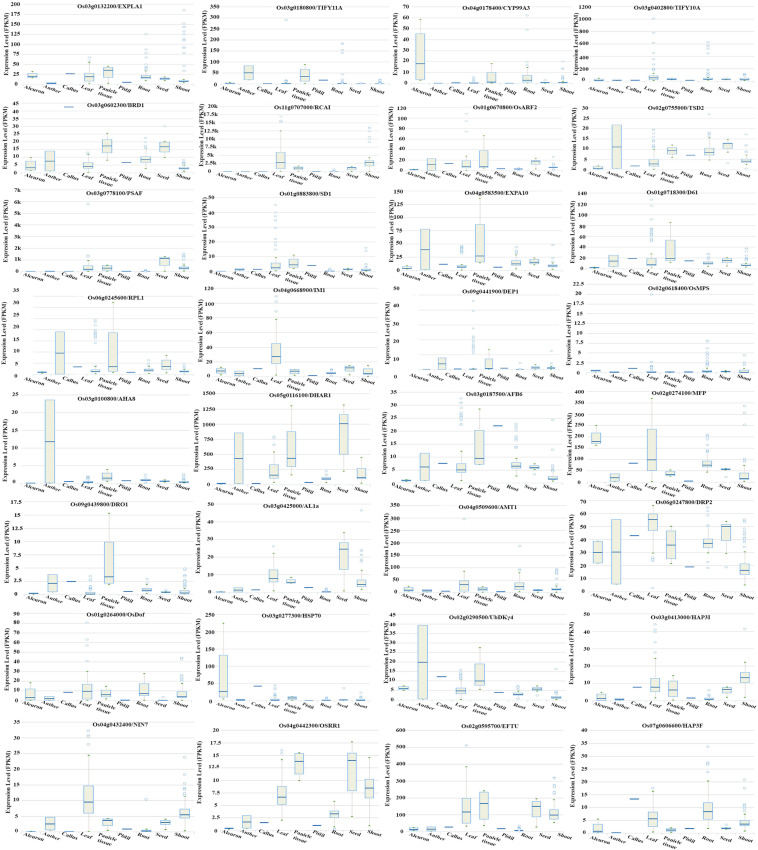
Tissue/state specific expression of phenotype-associated N-responsive genes. N-responsive genes identified in our microarray (GSE12940, GSE20924, and GSE140257) were shortlisted based on their reported association with yield and phenotypic traits identified in this study and their expression pattern retrieved from the Rice Expression Database is shown. They are EXPLA1, TIFY 11A, CYP99A3, TIFY 10A, BRD1, RCA1, ARF2, TSD2, PSAF, SD1, EXPA10, D61, RPL1, IM1, DEP1, OsMPS, AHAS, DHAR1, AFB6, MFP, DRO1, AL1a, AMT1, DRP2, OsDOF, HSP70, UbDKY4, HAP31, NIN7, OsRR1, EFTU, and HAP3F.

Further delineation of these 105 genes associated with the phenotype was done on the basis of their association with yield and N-response, as NUE is often defined in terms of yield/N. Out of the 105 genes associated with phenotype, a search at RicyerDB revealed that 54 genes were also associated with yield. These 54 genes were used for Venn selections with the 6,276 N-responsive genes identified in our transcriptomic studies (Pathak et al., 2020, GSE12940, GSE20924, and GSE140257). This revealed 34 N-responsive genes that were independently associated with yield as well as with the phenotypic traits shortlisted here for NUE (Supplementary Table S7). Out of them, 14 genes were reported as related to *plant height*, 11 to *flowering*, 7 to *root length*, 1 to *biomass*, 1 to *plant height* and *root length*, and 1 to *plant height* and *biomass* (Supplementary Table S7). These numbers are shown on the correlation map linking the various shortlisted phenotypic parameters, with respect to their urea response in our study (Figure 7). It clearly reveals that the identified NUE parameters span all the crucial stages of plant growth, indicating the physiological basis for phenology. A search for the known expression patterns of these 34 genes was carried out on the Rice Expression Database, which provides RNAseq data spanning nine tissues from 284 experiments (Xia et al., 2017). Most of them were found to be differentially expressed in terms of tissues or developmental stages, including few associated with the phenotype and/or yield such as RCA1, TSD2, EXP10, MFP, ABF6, DHAR1, and DRP2 (Figure 8). In view of their N-responsive expression in our microarray studies and reported association with yield and NUE phenotype, they emerge as candidate genes for NUE for further validation.

## Discussion

A major challenge for biological improvement of NUE in any crop has been the incomplete characterization of the phenotype and genotype for NUE, as is the lack of low-input screening programs (Mandal et al., 2018; Raghuram and Sharma, 2019) with rare exceptions (Rao et al., 2018). Rice is an excellent target crop to improve NUE, but the characterization of its phenotype is neither comprehensive nor complete, despite considerable progress, as detailed in the Introduction. Considering that developed countries use ammonium nitrate as fertilizer while developing countries mainly use urea, simultaneous phenotypic assessments with different forms of N are lacking to identify parameters of NUE that are common or unique to various forms of N. A major challenge in field phenotyping is the lack of precise control of N-form/dose in the soil and the difficulty to distinguish between intra-varietal and inter-varietal variation. Moreover, field experiments can either monitor many parameters in a few varieties or a few parameters in many varieties, making the identification of few critical parameters a crucial requirement for large scale screening of the germplasm.

The present study is a comprehensive life cycle analysis of 25 phenotypic parameters using two groups of rice genotypes grown in 32 replicates in the green-house with nitrate or urea as the sole N source at normal or low doses (four treatments) and field validation of two contrasting genotypes for NUE. The measured phenotypic parameters were chosen from those most commonly reported in literature, including the generic listing of rice traits by IRRI-IBPGR (1980). The rice genotypes of ssp. indica were chosen in two groups of 3 each, based on their contrasting germination rates and crop duration (early or late genotypes), with corresponding differences in yield and NUE as we reported earlier (Sharma et al., 2018). The experimental design minimized internal variation within each genotype by using seeds of modal weight (Sharma et al., 2018). External variation was minimized by using: (a) nutrient-depleted sand (Sharma et al., 2019) to eliminate the roles of soil nutrients and microbial N-transformations; (b) defined sterilized media with known concentrations of either nitrate or urea as the sole N source; (c) controlled temperature, humidity and light. This design enabled the identification of even subtle differences in the effect of N form and dose on various parameters and genotypes.

### Phenology of N Response

Yield is often associated with the NUE phenotype, as NUE is commonly defined in terms of yield per unit N. However, gross grain yield (measured as grain weight per plant or 1000 grain weight) as a distinctive inheritable property of each genotype seems to work differently from individual seed weight. Seeds of modal weight (±0.5 mg) were chosen for physiological evaluation of N response, as it was reported earlier that seed weight contributes to variation in germination, respiration, flowering time and yield in rice (Sitaramam et al., 2008). However, the next generation seeds fully restored their normal distribution of seed weights (Figure 1A), though their overall lower weights could be due to the lower light intensity in the green-house as compared to the field (Chen et al., 2019). We showed earlier that seeds of modal weight also vary in their rates of germination, which is further influenced by N (Sharma et al., 2018). The genetically enforced restoration of variation in seed weight and germination could be an evolutionary adaptation for survival in uncertain nutritional and growth environments. Nevertheless, modal weight varies between genotypes and we earlier demonstrated its utility in discerning subtle differences in the germplasm while phenotyping for yield (Sitaramam et al., 2008) and NUE (Sharma et al., 2018).

Phenotyping relies on genetically enforced variation in measurable traits in the germplasm. The coefficient of variation (CV) is a mean-standardized measure of variation, which is used to quantify and compare the variation of phenotypic traits (Pélabon et al., 2020). Our plots of CV against the time of measurement clearly revealed that variation in different parameters increased from vegetative to reproductive phase as well as depending on N-source and dose (Figures 1B,C). The low variance in seed germination and vegetative growth could be a reflection of the modal weights of seeds used. But it did not last beyond flowering, as a huge increase in variation in most yield parameters resulted in the restoration of variance in seed weight distribution in the next generation.

The higher reproductive variation in normal N levels (Figure 1B) relative to low N levels (Figure 1C) has implications for NUE, which is a derivative of reproductive performance (grain yield/N). High variation for reproductive parameters was reported in rice (Tiwari et al., 2019), but not in the context of N-response or NUE, while variation in chlorophyll has been recently linked with NUE (Jia et al., 2020). The restoration of variation from vegetative to reproductive phase is an unprecedented observation that prompts us to recommend the use of seeds of modal weight to control internal variation in vegetative parameters while phenotyping for any quantitative trait.

Principal component analysis segregated vegetative parameters and clustered the NUE-related vegetative parameters with reproductive parameters (Figure 2). The clustering of the *germination* (G) and *flowering* (R1) with the reproductive parameters is an important observation in line with our previous findings on their association with yield (Sitaramam et al., 2008), crop duration, and NUE (Sharma et al., 2018). These findings were further validated here by PLS-DA (Supplementary Figure S4). Thus, the qualitative differences in physiological variance in terms of vegetative and reproductive parameters are as important as their quantitative differences in their response to variation in the source and dose of N.

MANOVA confirmed that the effect of N was significant when parameters were bunched into vegetative and reproductive groups (*P* < 0.0001), regardless of the magnitude of the effect itself. Two-way ANOVA using treatments and genotypes helped to identify 20 individual parameters in which N effect was significant. The overall effect of low N was to delay germination and flowering and up-regulate yield parameters, while down-regulating all other vegetative parameters (Figure 3). These findings are consistent with earlier findings that showed low vegetative biomass under low N conditions (Chen et al., 2013). We showed earlier that germination and flowering can predict rice yield (Sitaramam et al., 2008), while germination time and crop duration constitute phenotypic parameters for NUE (Sharma et al., 2018). Others linked NUE in rice with root growth (Morita et al., 1988; Xu et al., 2012; Yang et al., 2012; Li et al., 2015; Peng et al., 2015; Rogers and Benfey, 2015; Kiba and Krapp, 2016; Nguyen et al., 2016; Steffens and Rasmussen, 2016; Xie et al., 2017; Rao et al., 2018; Duque and Villordon, 2019), *chlorophyll* (Ladha et al., 2007; Thind and Gupta, 2010; Mathukia et al., 2014; Jia et al., 2020), dense and erect panicle (Sun et al., 2014), *panicle number* (Jia et al., 2020), *plant height* (Sitaramam et al., 2008; Kumar et al., 2014; Nguyen et al., 2016; Selvaraj et al., 2017), and *biomass* (Nguyen et al., 2016; Selvaraj et al., 2017).

But none of them specifically claimed them as adequate phenotypic parameters for NUE, nor evaluated them in comparison with all other physiological parameters. Our comprehensive physiological evaluation of 25 parameters, more than any other study so far (Devi and Sumathi, 2011; Selassie, 2015; Anil et al., 2018; Rao et al., 2018), identified 20 of them as significantly N-responsive including root growth, plant height and biomass cited above for further shortlisting and delineation of the NUE phenotype (Table 2).

### Phenotype Prediction by Interactive Networks

An important aspect of phenotyping for a complex quantitative trait like NUE is to find the interacting parameters that work together. Bi-dimensional networks based on correlation matrix allow segregation of clusters of variables and visualization of their connections (Ursem et al., 2008). This approach popular in genomics and has been recently explored for plant phenotyping (Oladosu et al., 2018; Baye et al., 2020; He et al., 2020), though never applied to NUE phenotyping so far. Therefore, we constructed a network of all the significantly correlated measured parameters (Figure 4). The most connected parameters in all three N regimes were *germination*, *plant height before/after harvest*, *panicle weight*, *flowering* and grain yield related parameters, while the least connected parameters were leaf-related vegetative parameters, with the only exception of *filled grain weight* in the nitrate regime. This is consistent with our earlier report on germination time (Sharma et al., 2018) and also with several other parameters that emerged so far from this study (Figures 3, 4).

Interestingly, urea regime revealed two distinct networks (Figure 4C), with *stem thickness* and all the leaf-related parameters including *chlorophyll* clustered on the right, while all parameters contributing to reproductive yield clustered on the left along with *germination* and *flowering*, *plant height*, *root length*, and *biomass*. These are the only measured vegetative parameters that contribute to the phenotype of NUE, as we confirmed later through correlation studies with yield in low N (Supplementary Figure S2) and NUE (Table 1 and Supplementary Figure S3). More importantly, these parameters in urea are very similar to the most connected ones in the nitrate network or combined network, indicating the possible universal biological interactions/mechanisms that underlie N-response and NUE. This is a novel visualization of interacting parameters for NUE phenotype and may help in phenotyping other complex traits, apart from revealing their connections with yield.

### Phenotype Prediction by Feature Selection/Ranking

Germplasm screening for NUE requires the shortlisting of a minimal set of phenotypic traits, especially of vegetative parameters, as reproductive yield is inherent to the agronomic definition of NUE. Feature selection is a well-known computational method to rank and identify the best among the many available Features (Abeel et al., 2009), though never used in plant phenotyping. Therefore, we used it to rank and identify the best among the measured vegetative parameters (and flowering) without observational bias in the present study. This allowed shortlisting of 11 out of the 17 growth parameters from germination to flowering (Supplementary Table S4). The top eight parameters among them correlated positively with N-responsive yield (Supplementary Figure S2) and NUE (Table 1 and Supplementary Figure S3). This is an independent validation of the eight vegetative parameters revealed by ANOVA (Table 2). These are also the same eight vegetative parameters which displayed very narrow range of variation (Figures 1B,C), attributable to using seeds of modal weight, indicating its methodological importance in phenotyping.

### Segregation and Shortlisting of Parameters for NUE Phenotype

There was no phenotypic distinction between N-response and NUE in literature. Our study of 25 parameters has identified 20 parameters as significantly N-responsive, including 17 growth parameters from germination to flowering, out of which six were segregated as contributing to NUE in both nitrate and urea and two others as contributing to NUE in urea only. The correspondence between multiple approaches to data analysis viz PCA (Figure 2), ANOVA (Figure 3), network analysis (Figure 4), correlation with grain yields (Supplementary Figure S2), NUE (Supplementary Figure S3), PLS-DA (Supplementary Figure S4), and Feature selection (Supplementary Table S4) reflects the robustness and validity of our novel methodology (Figure 5). This novel flowchart could also help in the phenotyping of other complex traits in rice and other plants.

A closer examination of the eight NUE parameters in terms of the % effect of N on each of them and their correlation with total grain yield and NUE (Supplementary Table S8) revealed that the % N effect on yield was similar to % N effect on *germination* in both nitrate and urea regimes, and both the effects were amplified on NUE. In other words, even small changes in the identified phenotypic parameters contribute to major changes in NUE, even if they are not always discernible in terms of yield alone. Moreover, we obtained the most rigorous biological evidence to date that in addition to yield-related parameters, 6 phenotypic parameters for NUE are common to nitrate and urea, unknown in any crop so far. Such parameters that are independent of N-form are of potential universal value to improve crop NUE, considering the different forms of N used as fertilizer and their dynamic mixtures in different farm soils. Our experimental design had a crucial role in reliably capturing several such phenotypic nuances in the green-house and is compatible with automated phenomics.

Overall, our green-house findings clearly show that low N levels (1.5 mM) improve yield and NUE by delaying seed germination and flowering and limiting all other vegetative growth parameters. Normal N levels (15 mM) favor vegetative growth but limit grain yield parameters and reduce NUE. It is known that excessive N doses are not conducive to nutrient absorption (Yuan and Chen, 2015), accumulation and translocation of non-structural carbohydrates and do not promote yields (He et al., 2001), but only *biomass* (Singh et al., 2014) and even reduce yield (Shi et al., 2020). On the other hand, reduced N doses resulted in increased activities of enzymes that increased the remobilization of stem starch and non-structural carbohydrates, thus increasing grain filling and harvest index in rice (Li G. et al., 2018). Further, high NUE was associated with better photosynthetic performance in rice (Ju et al., 2015).

Literature did not recognize the broadly opposite response of vegetative and reproductive parameters to N dose. Considering that vegetative growth depends mostly on cell elongation (Löfke et al., 2015), whereas reproductive growth mostly relies on meristematic cell division, they may point toward the physiological mechanism for NUE. Our earlier energetic basis for drought or water use efficiency (Sitaramam et al., 2007) and yield itself (Sitaramam et al., 2008) may also be of relevance for NUE, as indicated by our recent findings on the role of respiration in N-responsive germination and NUE (Sharma et al., 2018). Our field evaluation of two rice genotypes (Figure 6) clearly validated their selection on a phenotypic basis and revealed the superior yield and NUE indices of the late-germinating, late-flowering and long-duration genotype, Panvel1, relative to Nidhi, the early germinating, early flowering, and short-duration genotype. This also validates our greenhouse phenotyping that avoids the uncertainties of field phenotyping and enables automated phenomics.

### From Phenotype to Genotype for NUE

Functional genomics provided us with thousands of genes involved in N-response (Mandal et al., 2018; Raghuram and Sharma, 2019), including our own data on rice (Pathak et al., 2020, GSE12940, GSE20924, and GSE140257). Our delineation of the parameters constituting the NUE phenotype fills this gap. Their visualization in terms of the growth physiology clearly revealed that all crucial stages of plant growth are linked to NUE and/or yield (Figure 7). Further, our identification of 105 genes associated with the NUE phenotype from literature (Supplementary Table S6), of which 54 were associated with yield, enabled the identification of 34 of them as N-responsive from our microarray data (Supplementary Table S7).

We found that all these 34 N-responsive genes associated with our NUE phenotype as well as with yield in rice were differentially expressed in the Rice Expression Database (Figure 8). The role of a few of them in NUE has been validated. For example, overexpression of *OsAMT 1.1* was reported to increase NH4^+^ uptake and grain yield under suboptimal and optimal N conditions, as well as NUE (Ranathunge et al., 2014). Overexpression of *OsRDD1* was shown to increase nutrient uptake and accumulation, N responsiveness and grain productivity (Iwamoto and Tagiri, 2016). The overexpression of *OsDEP1* has also been reported to improve NUE (Zhao et al., 2019). While most of the 34 genes we reported here remain to be validated for their role in NUE, their attraction stems from the above reports that corroborate our comprehensive approach from phenotype to genotype. Our list also opens the opportunity for investigating the underlying molecular mechanisms, apart from identifying the best combination of genes that deliver the maximum improvement in a multi-genic quantitative trait like NUE.

## Conclusion

Our comprehensive life cycle analysis of rice genotypes for 25 phenotypic parameters identified 20 N-responsive parameters, from which a subset of eight NUE parameters was segregated and two genotypes chosen on such phenotypic basis were validated in the field. Six of these eight parameters work well for both urea and nitrate, making them potentially universal NUE parameters. Our comprehensive phenotyping for NUE in rice may be of methodological relevance to other plants, and possibly to other nutrients. Our green-house-based phenotyping is amenable to automation for phenomics. The correspondence between the PCA, network, Feature selection, correlation and PLS-DA makes it a novel and robust approach for phenotyping other complex traits. The identification of associated genes and shortlisting of 34 candidate genes for NUE by linking the phenotype with the genotype opens up opportunities for their validation and further investigation into the underlying mechanisms, as well as for crop improvement.

## Data Availability Statement

All data are available within the manuscript or Supplementary Material, including data extracted from GEO (accession numbers: GSE12940, GSE20924, GSE140257, and GSE122596) or gene lists compiled from literature (Supplementary Table S5).

## Author Contributions

NS performed most of the experiments, data analysis, and wrote the first draft. VS helped in early green house experiments. NP, DS, CN, and SK performed the field experiment. AJ performed the Feature selection analysis. VS helped in data analysis. RP provided the statistical suggestions. NR helped in the planning, mentoring, and supervision of the experiments, data interpretation, and manuscript preparation. All the authors contributed to the article and approved the submitted version.

## Conflict of Interest

The authors declare that the research was conducted in the absence of any commercial or financial relationships that could be construed as a potential conflict of interest.

## Footnotes

1 https://shigen.nig.ac.jp/rice/oryzabase/

2 https://bioinfogp.cnb.csic.es/tools/venny/

3 http://linux1.softberry.com/berry.phtml?Topic=protcomppl&group=programs&subgroup=proloc

## Supplementary Material

Supplementary Figure 1 Green-house experimental design.

Supplementary Figure 2 Correlation of phenotypic parameters with yield at low N.

Supplementary Figure 3 Correlation of nine shortlisted phenotypic parameters with NUE.

Supplementary Figure 4 PLS-DA of the effect of N on 25 phenotypic parameters in five rice genotypes.

Supplementary Figure 5 Predicted protein localization of 105 genes related to shortlisted phenotypic parameters.

Supplementary Table 1 Dates of sowing, planting and harvest for Rabi 2017-18 and Kharif 2017 and 2018.

Supplementary Table 2 Individual PCA scores.

Supplementary Table 3 Mean values of the measured 25 phenotypic parameters in nitrate/urea sources and normal (15 mM) or low (1.5 mM) doses.

Supplementary Table 4 Mean values along with standard deviation and standard errors for each of the N treatment of the measured 25 phenotypic.

Supplementary Table 5 Ranking of vegetative phenotypic parameters of N response by Feature selection method.

Supplementary Table 6 Genes associated with shortlisted phenotypic parameters.

Supplementary Table 7 N responsive genes listed for shortlisted phenotypic parameters and associated to yield.

Supplementary Table 8 Impact of normal N *vis-a-vis* low N on each of the vegetative parameters correlated with yield and NUE.
