# Supplementary figures and images for: Nitrogen Use Efficiency Phenotype and Associated Genes: Roles of Germination, Flowering, Root/Shoot Length and Biomass
Source: Front Plant Sci. 2021 Jan 20;11:587464. doi: 10.3389/fpls.2020.587464 (PMC7855041; doi:10.3389/fpls.2020.587464)

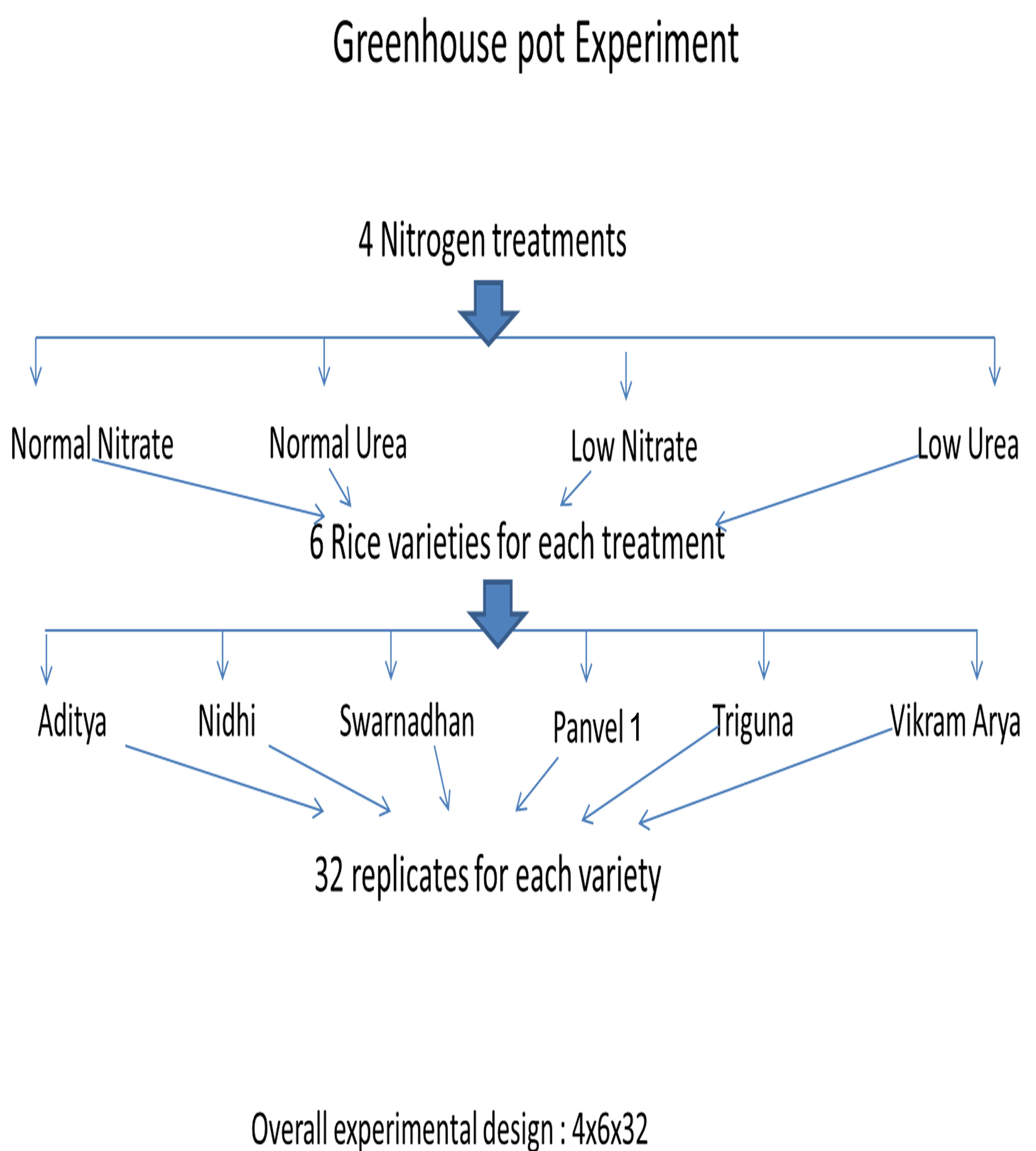

Supplement: Supplementary Figure 1 — Green-house experimental design. [file Image_1.TIF]

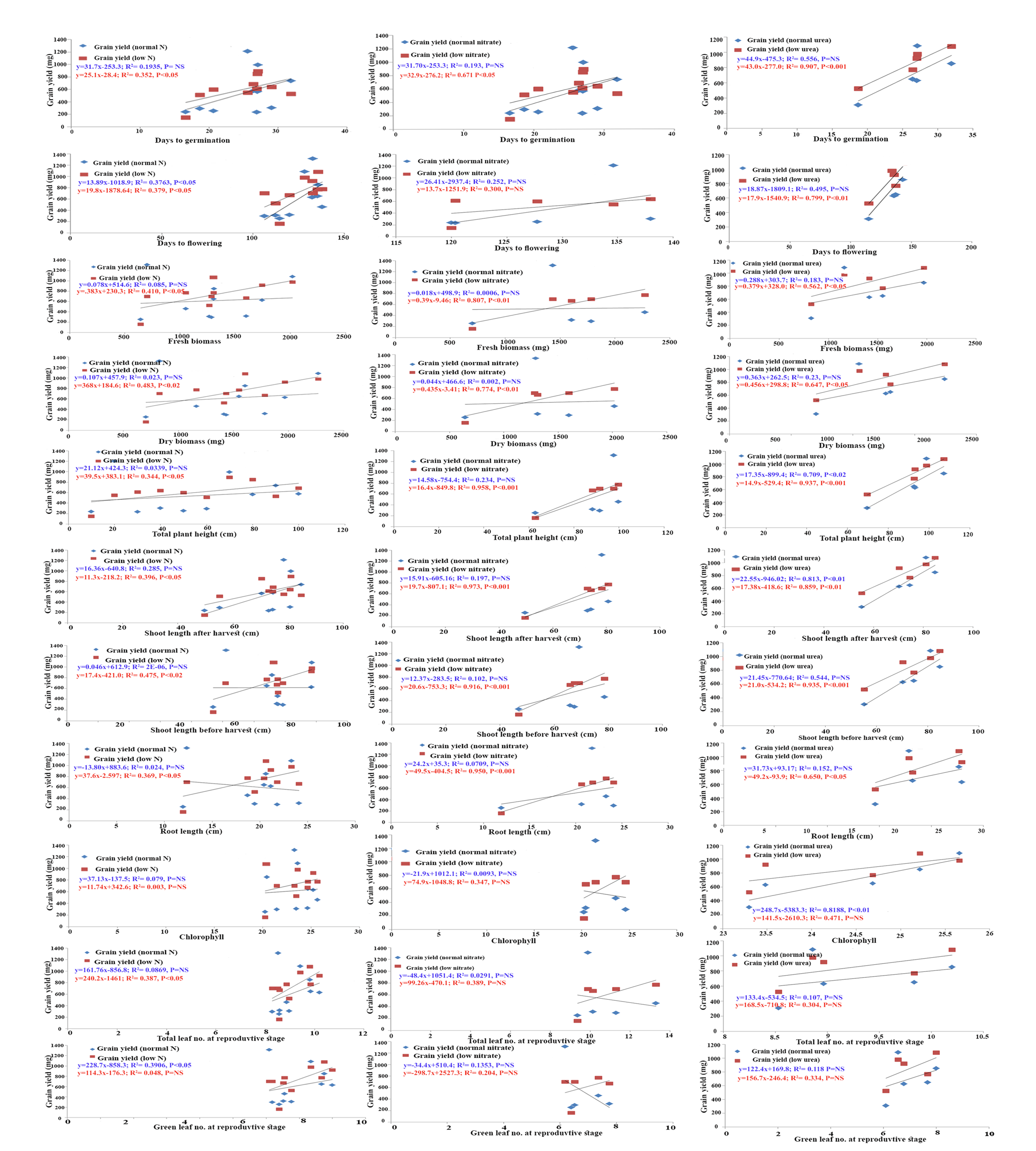

Supplement: Supplementary Figure 2 — Correlation of phenotypic parameters with yield at low N. [file Image_2.TIF]

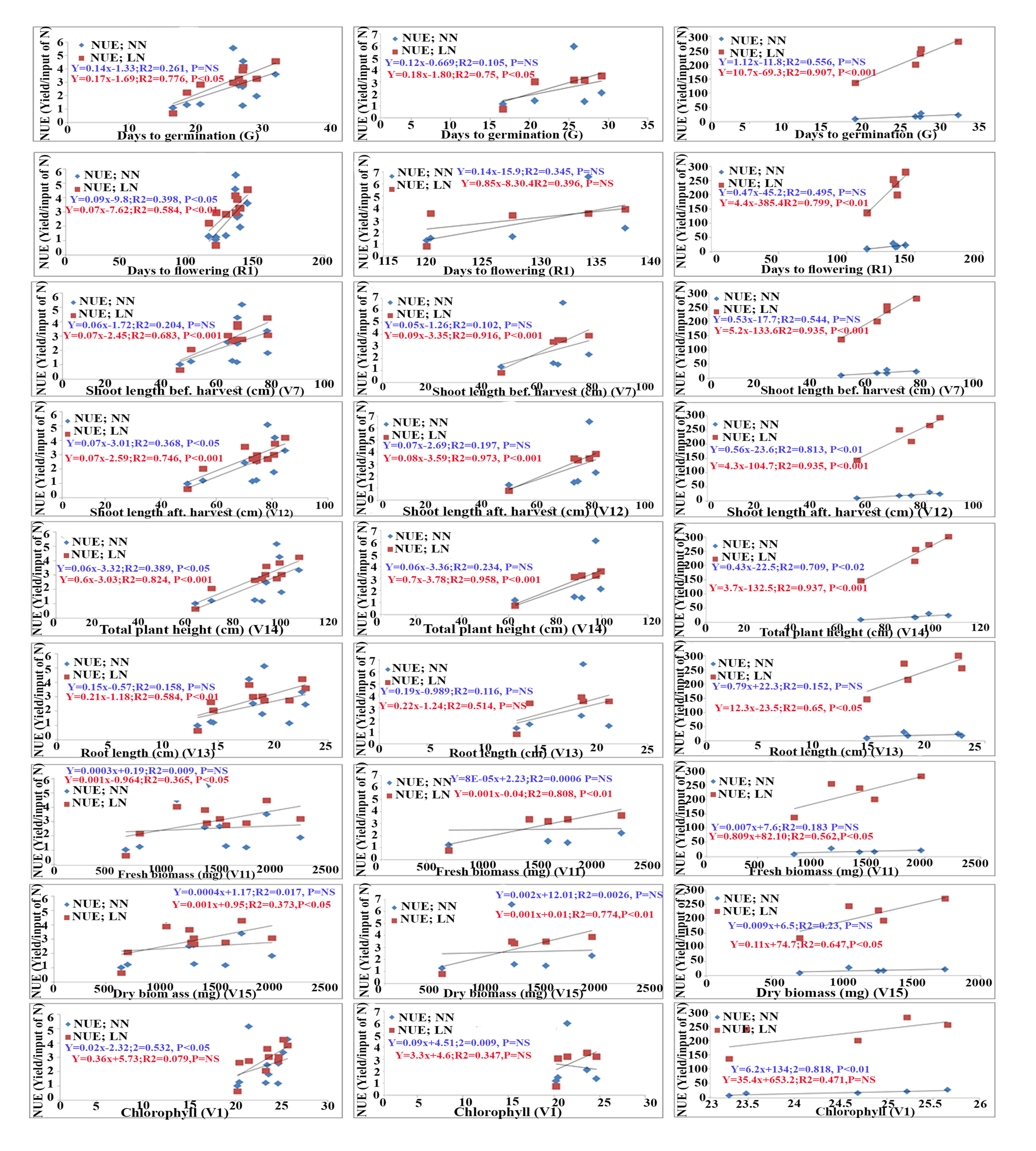

Supplement: Supplementary Figure 3 — Correlation of nine shortlisted phenotypic parameters with NUE. [file Image_3.TIF]

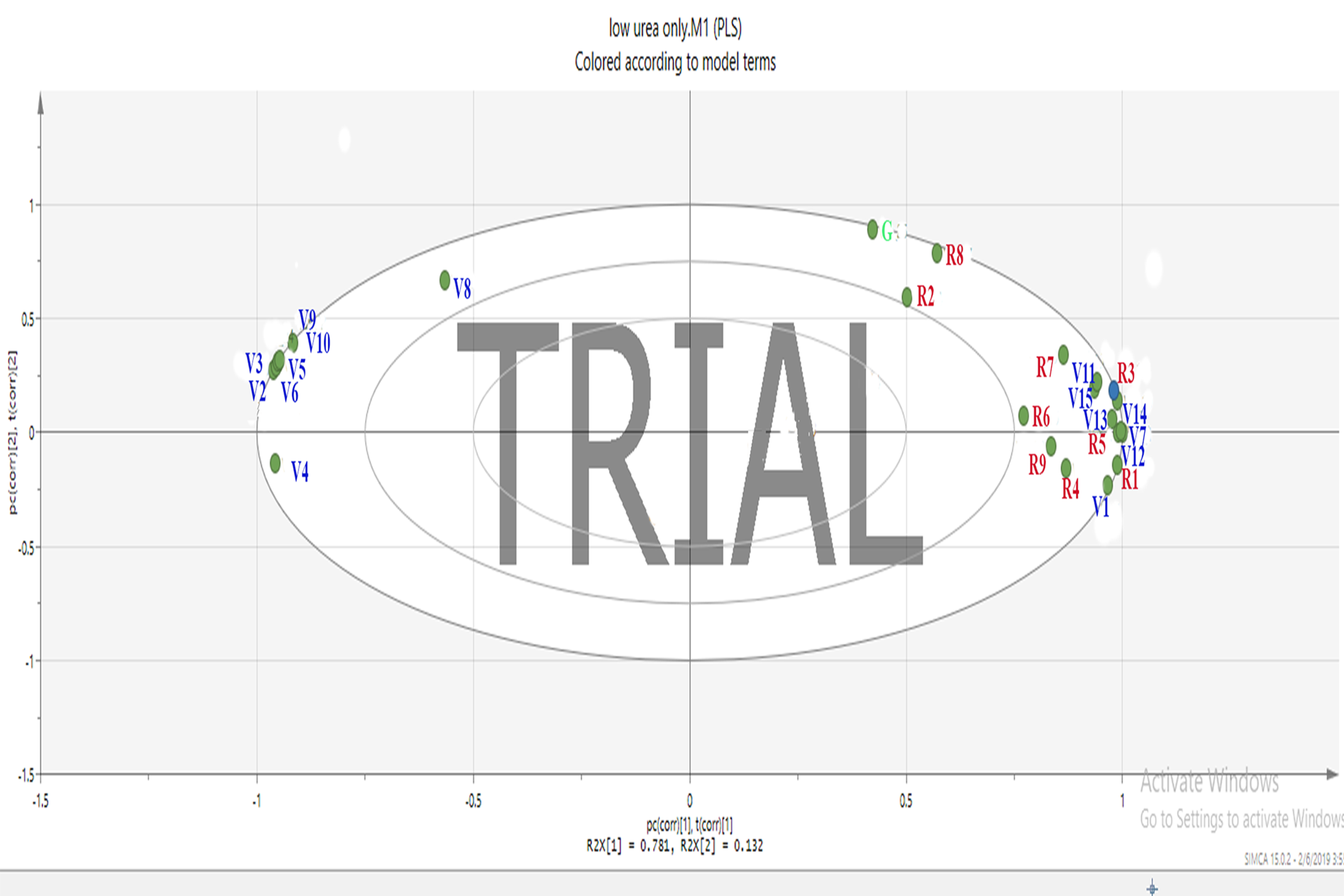

Supplement: Supplementary Figure 4 — PLS-DA of the effect of N on 25 phenotypic parameters in five rice genotypes. [file Image_4.TIF]

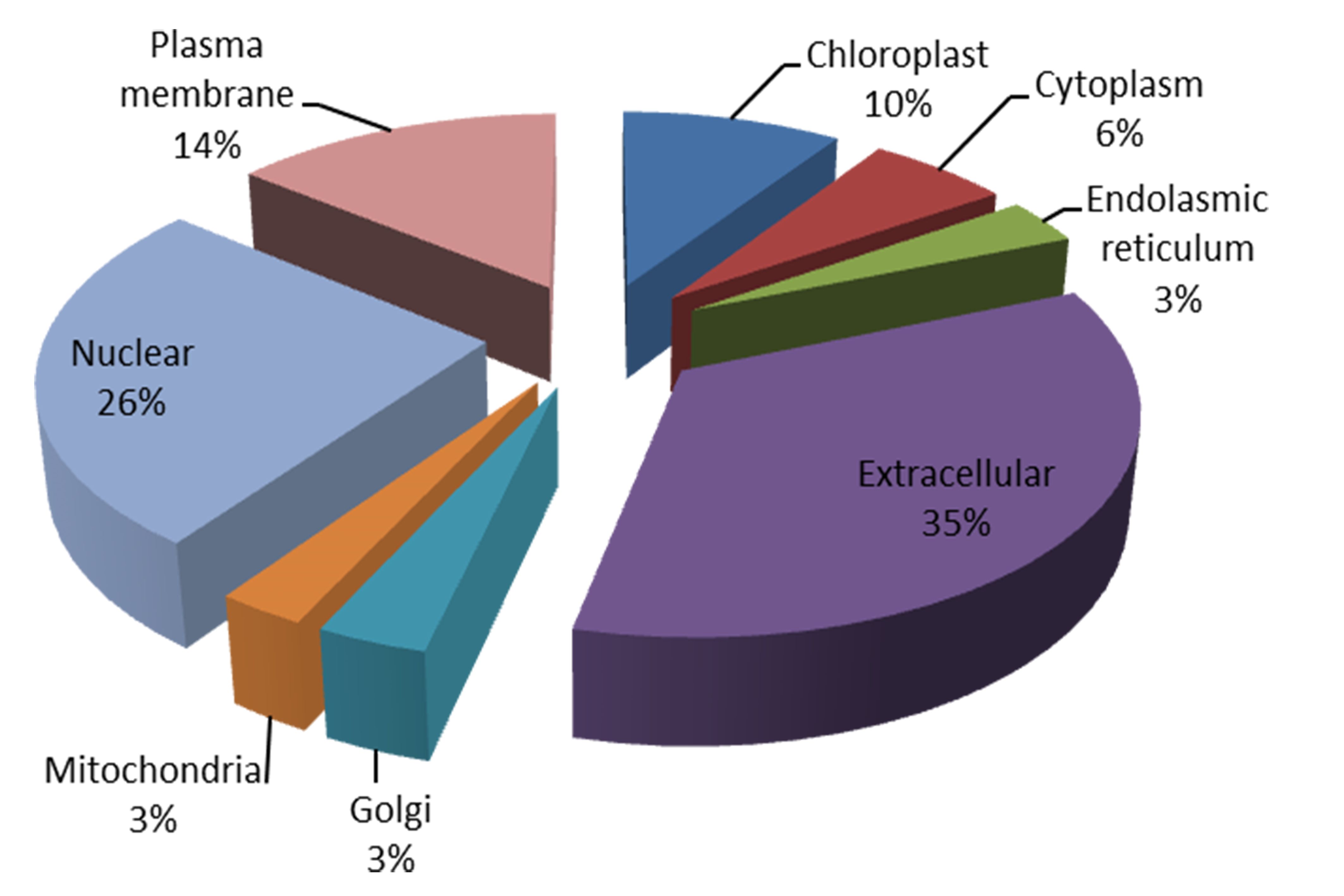

Supplement: Supplementary Figure 5 — Predicted protein localization of 105 genes related to shortlisted phenotypic parameters. [file Image_5.TIF]
